# Dietary Influence on Bacterial Vaginosis

**DOI:** 10.7759/cureus.93506

**Published:** 2025-09-29

**Authors:** Dhiya Ram, Pujita Julakanti, Nabiha T Atiquzzaman, Stephanie Nagy, Annie Y Lin, Marc M Kesselman

**Affiliations:** 1 Internal Medicine, Nova Southeastern University Dr. Kiran C. Patel College of Osteopathic Medicine, Davie, USA; 2 Rheumatology, Nova Southeastern University Dr. Kiran C. Patel College of Osteopathic Medicine, Davie, USA; 3 Nutrition, Nova Southeastern University Dr. Kiran C. Patel College of Osteopathic Medicine, Davie, USA

**Keywords:** bacterial vaginosis (bv), carbohydrate, diet, dietary influence, diet modification, gardnerella vaginalis, haemophilus haemolyticus vaginalis, probiotic interventions, vitamins and minerals

## Abstract

Bacterial vaginosis (BV) is a prevalent vaginal infection in women of reproductive age, characterized by a dysbiosis of the vaginal microbiota. A healthy vaginal environment is typically dominated by *Lactobacillus* species, which maintain a low pH that inhibits pathogenic bacterial growth. Emerging evidence suggests that diet may significantly influence the composition and function of the vaginal microbiota, offering a modifiable risk factor for BV. This systematic review examines the relationship between dietary patterns and BV risk, focusing on the effect of vitamins/minerals, fruits/vegetables, dairy/probiotics, carbohydrates/sugar, grains/fibers, and protein on vaginal health. It has been shown that deficiencies in vitamins A, C, E, and D may increase susceptibility to BV due to their roles in immune function, antioxidative defense, and vaginal epithelial health. Increased fruit and vegetable consumption, which provides these essential vitamins alongside phytochemicals such as betaine, correlates with a reduced risk of BV. Conversely, high glycemic diets appear to promote BV through increased oxidative stress. Whole grains and dietary fiber, rich in anti-inflammatory compounds and essential nutrients, support *Lactobacillus*-dominant microbiota and reduce BV risk. Additionally, obesity is associated with increased BV prevalence, suggesting a potential link between metabolic health and vaginal microbiota. The role of probiotics and dairy in fostering* Lactobacillus* colonization shows promise, while plant-based protein sources may further support vaginal health by reducing inflammation and maintaining pH balance. Despite these findings, limitations exist, including the need for longitudinal studies to determine causality, dose-dependent relationships, and the impact of dietary interventions across diverse populations. Future research should explore the efficacy of specific dietary nutrients and probiotics, particularly in high-risk groups, to develop targeted dietary recommendations for BV prevention.

## Introduction and background

Bacterial vaginosis (BV) is a prevalent vaginal infection occurring in women of reproductive age due to dysbiosis of the vaginal microbiota. A healthy vaginal microbiome is typically dominated by *Lactobacillus* species and has a low pH. This imbalance in BV is characterized by decreased *Lactobacillus* species and increased anaerobic bacteria, such as *Gardnerella* species [1]. Common symptoms due to this imbalance include vaginal odor, discharge, and discomfort. Persistent infection is associated with an increased risk of pelvic inflammatory disease (PID), preterm birth, sexually transmitted infections (STIs), and infertility [2]. Although BV is highly prevalent around the world, affecting as many as 60% of females per country, effective long-term management strategies are limited, indicating the necessity for the exploration of modifiable risk factors, such as diet [2].

Risk factors of BV include douching, smoking, sexual activity, antibiotic use, socioeconomic status, and hygiene, all of which can cause an imbalance in the vaginal microbiota, thus increasing the risk for BV [3]. Another potential risk factor that has been recently identified due to its role in contributing to systemic inflammation is obesity [3]. Moreover, due to increased susceptibility to STIs such as human immunodeficiency virus (HIV), BV, although not classified as an STI itself, is a significant issue in public health [4]. In addition, with the imbalance occurring in reproductive-age women, pregnant women are at higher risk for BV [2]. Other comorbidities of BV include endometriosis and post-surgical infections, on top of PID and fertility issues [5]. Dietary intake can modulate systemic inflammation and the vaginal microbiome through mechanisms involving immune regulation, oxidative stress, and metabolic health. These pathways may underlie potential associations between nutrition and BV risk [5].

In studies conducted among various ethnic groups, including African Americans, those suffering from BV were 1.5 to over 3.0 times more likely to have suboptimal diets [5]. Due to the implications of BV, it is essential to research prevention strategies. Recent research has shown promising results with the relationship between vaginal health and diet, suggesting that deficiencies in certain nutrients can increase the risk of contracting BV. Recognizing the role of specific dietary components, dietary patterns, and specific micronutrients may be beneficial in creating a proactive prevention and/or treatment plan. This systematic review aims to explore relationships between specific dietary patterns and provide evidence-based recommendations for reducing BV risk.

## Review

Methodology

An initial literature search was conducted on Google Scholar to gain a brief overview of the topic, after which certain keywords were sought from relevant articles. With BV being the pathology of interest, the keyword “Bacterial vaginosis” was searched across abstracts, keywords, and titles, alongside second-level keywords, including “bacterial vaginitis” OR “non specific vaginitis” OR “nonspecific vaginitis” OR “vaginosis, bacterial”. Next, keywords such as “Gardnerella vaginalis” OR “Corynebacterium vaginale” OR “Corynebacterium vaginalis” OR “Haemophilus haemolyticus vaginalis” OR “Haemophilus hemolyticus vaginalis” OR “Haemophilus vaginalis” OR “hemophilus vaginalis” were searched for across abstracts, keywords, and titles due to the known association between BV and these microbial species. Articles from these two keyword searches were further connected via the Boolean operator “OR.” Finally, the keyword “Diet” was searched across abstracts, keywords, and titles, alongside second-level keywords, including “diet influence” OR “diet regimen” OR “diet surveys” OR “dietary effect” OR “dietary influence” OR “dietary survey” OR “dietary surveys” OR “dieting” and were connected with the previous article findings via the Boolean operator “AND.” The search strategy was replicated across three databases, namely, EMBASE, Ovid MEDLINE, and Web of Science on November 19th, 2024. The initial search yielded 163 articles.

**Figure 1:**
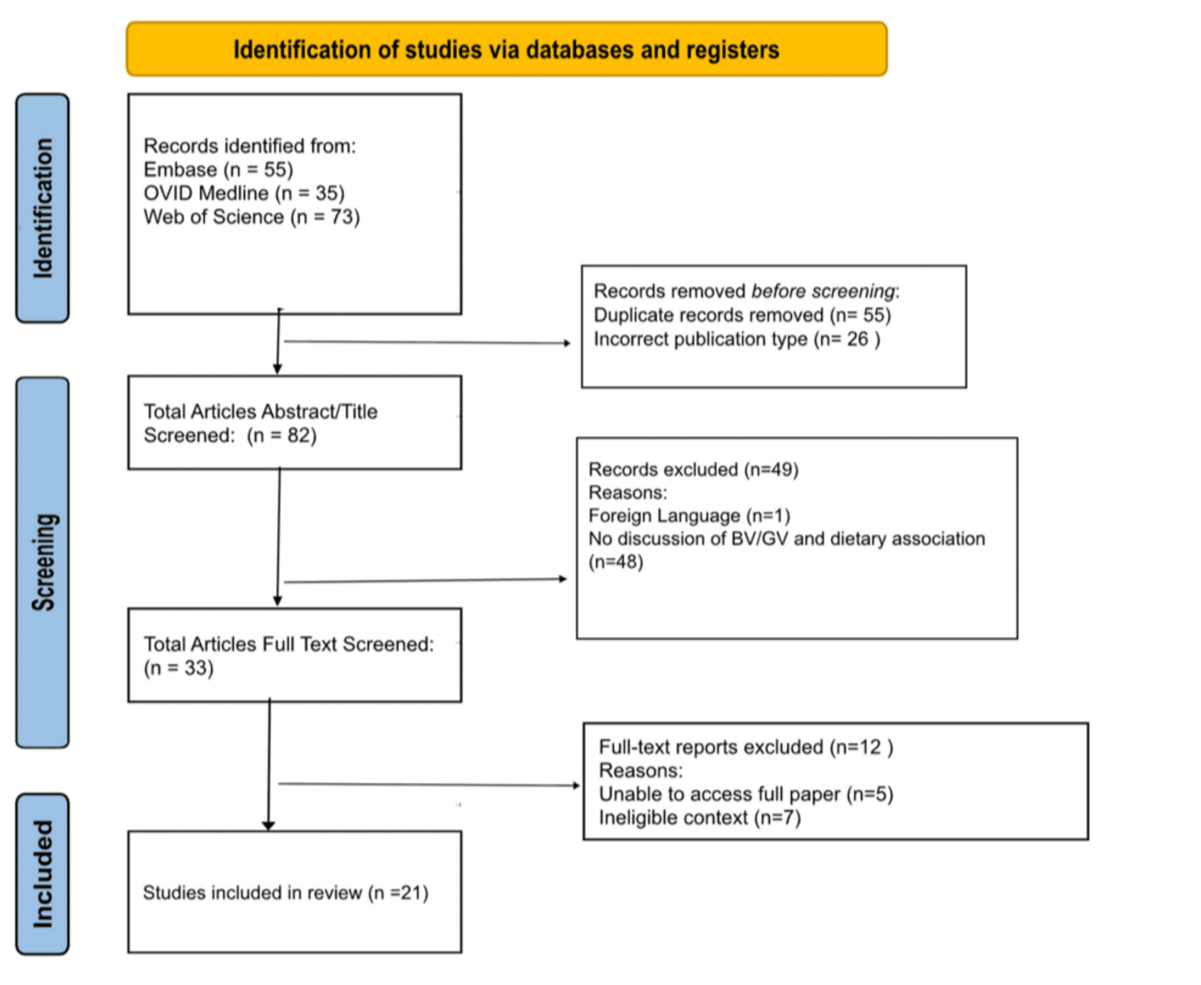
Preferred Reporting Systems for Systematic Reviews and Meta-Analyses (PRISMA) flowchart of the selection procedure.

Before screening and the article selection process, EndNote 21 was used to remove duplicates, after which 108 articles were imported to Rayyan. In total, 26 articles were immediately excluded for being incorrect publication types for systematic reviews. The remaining 82 articles went through Tier 1 title and abstract screening, after which 49 articles were excluded, leaving 33 articles. Further, 12 more articles were excluded after full-text screening, leaving 21 articles to be included in the scoping review. The Preferred Reporting Items for Systematic Reviews and Meta-Analyses (PRISMA) flowchart details the study’s article screening and selection process (Figure 1) [6].

Results

Data were extrapolated from the final 21 papers and charted independently by each of the three reviewers. The variables utilized in the form included aspects of the study design and nutrition category. Table 1 shows the 21 articles analyzed for the study design, nutrition category, and number of subjects in the selected studies [7-21].

**Table 1:** Analysis of selected studies. BMI: body mass index

Study	Study design	Nutrient category	Number of subjects
Bisanz et al., 2015 [7]	Clinical trial	Vitamins and minerals	56
Noormohammadi et al., 2022 [8]	Case-control study	Vitamins and minerals, protein, diet, BMI	295
Rosen et al., 2021 [9]	Cross-sectional study	Vitamins and minerals, fruits and vegetables, grains and fiber	634
Tuddenham et al., 2019 [10]	Cross-sectional study	Vitamins and minerals	104
Song et al., 2020 [11]	Cross-sectional study	Vitamins and minerals, fruits and vegetables, diet, BMI	26
Noormohammadi et al., 2022 [12]	Case-control study	Vitamins and minerals, fruits and vegetables, carbohydrates and sugar, protein, diet	294
Khademian et al., 2024 [13]	Case-control study	Vitamins and minerals	294
Akoh et al., 2017 [14]	Cross-sectional study	Vitamins and minerals, BMI	158
Noormohammadi et al., 2022 [15]	Case-control study	Fruits and vegetables, protein, BMI	294
Vargas-Robles et al., 2024 [16]	Cross-sectional study	Fruits and vegetables	48
Fettweis et al., 2014 [17]	Cross-sectional study	Dairy and probiotics	1,684
Laue et al., 2018 [18]	Randomized clinical trial	Dairy and probiotics	36
Happel et al., 2017 [19]	Cross-sectional study	Dairy and probiotics	-
Oerlemans et al., 2022 [20]	Clinical trial	Dairy and probiotics	52
Gabriela et al., 2023 [21]	Cohort study	Dairy and probiotics	516
Houttu et al., 2022 [22]	Randomized clinical trial	Dairy and probiotics	439
Noormohammadi et al., 2022 [23]	Case-control study	Carbohydrates and sugar, grains and fiber	301
Dall'Asta et al., 2021 [24]	Cohort study	Carbohydrates and sugar, grains and fiber, protein, BMI	24
Miller et al., 2024 [25]	Cohort study	Carbohydrates and sugar	-
Sun et al., 2022 [26]	Randomized clinical trial	Grains and fiber	303
Shivakoti et al., 2020 [27]	Cross-sectional study	Grains and fiber	104

**Figure 2:**
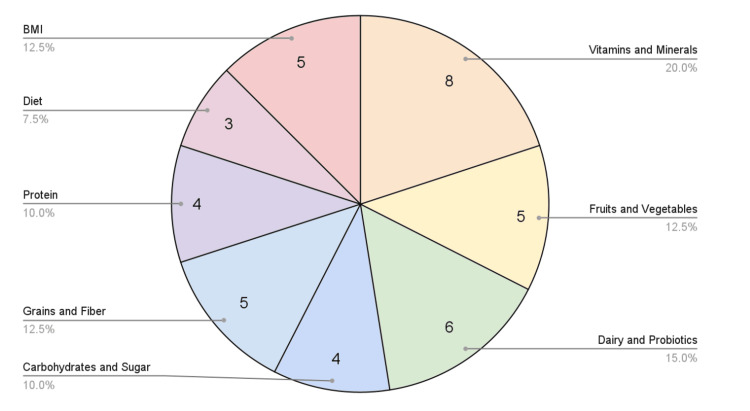
This pie chart summarizes the findings of the studies recorded in Table 1 and groups the nutrient categories based on the number of papers identified in this review. BMI: body mass index

Figure 2 shows a pie chart of the most prevalent evidence-based nutrient categories and their relationship with BV based on the number of papers found in this scoping review of recent literature: vitamins and minerals with eight articles, fruits and vegetables with five articles, dairy and probiotics with six articles, carbohydrates and sugar with four articles, grains and fiber with five articles, protein with four articles, diet with three articles, and BMI with five articles [7-27].

Vitamins and Minerals

Table 2 shows the pertinent findings of articles that analyze the relationship between vitamins and minerals and BV [7-14].

**Table 2:** Pertinent findings of studies describing the role of vitamins and minerals in BV. BV: bacterial vaginosis

Study	Pertinent findings
Bisanz et al. [7]	Increased calcium levels from probiotic consumption correlates with increased vaginal microbial diversity
Noormohammadi et al. [8]	BV affected women had lower calcium supplement use than controls
Rosen et al. [9]	Increased intake of vitamin D associated with increased *Lactobacillus crispatus* in the vaginal microbiome in black women
Tuddenham et al. [10]	Low betaine dietary intake correlated with higher odds of having BV
Song et al. [11]	Low vitamins A, C, and E and β-carotene are associated with increased risk of BV
Noormohammadi et al. [12]	Excess sodium consumption raises risk of contracting BV
Khademian et al. [13]	Higher intake of dietary phytochemicals lowers risk of BV
Akoh et al. [14]	Identified an increased prevalence of BV in adolescents who were deficient of several essential micronutrients

Several vitamin and mineral deficiencies, such as low calcium and vitamin D, were found to be associated with an increased risk of BV [7-10]. Additionally, deficiencies in vitamins A, C, and E, as well as low beta-carotene and betaine, have also been linked to acquiring BV [10,11]. However, excess sodium consumption appeared to increase the odds of developing BV [12]. A higher intake of dietary phytochemicals, on the other hand, was shown to lower the risk of BV, emphasizing the potential benefits of a plant-based diet [13]. Other minerals, such as zinc, selenium, and lutein, only showed borderline significant association with BV [10].

Results of a study performed specifically on pregnant populations demonstrate similar findings. The results of this study utilized estimated average requirement (EAR), a daily nutrient intake level estimated to meet the needs of 50% of healthy individuals in a specific group, as a measure of adequate nutrient intake [14]. Of 158 pregnant adolescents studied, the majority (70-90%) did not meet the EAR for several essential micronutrients. In this population, BV was found to be one of the four most commonly diagnosed infections [14].

Fruits and Vegetables

Table 3 shows the pertinent findings of articles that analyze the relationship between fruits and vegetables and BV [9,11,12,15,16].

**Table 3:** Pertinent findings of studies describing the role of fruits and vegetables in BV. BV: vacterial vaginosis

Study	Pertinent Findings
Rosen et al. [9]	Increased intake of fruits associated with increased *Lactobacillus crispatus* in the vaginal microbiome in black women
Song et al. [11]	Vegetarians demonstrated a higher vaginal microbial diversity when compared to nonvegetarians. (p = 0.004)
Noormohammadi et al. [12]	Vegetables linked to decreased odds of BV
Noormohammadi et al. [15]	A significant association found between the highest tertiale of vegetables and decreased BV odds in both crude and adjusted models
Vargas-Robles et al. [16]	Pregnant women with optimal vegetable intake demonstrated a vaginal microbiota dominated by *Lactobacillus crispatus*, leading to a decreased risk of BV

Multiple studies have highlighted the role of fruits and vegetables in preventing BV by emphasizing their potential to promote a healthier vaginal microbiota [9,11,12,15,16]. High vegetable intake was found to decrease BV risk, with odds of BV 72% and 66% lower with a vegetable intake of more than 415 g/day [12,15]. This finding supports a trend discovered in pregnant and nonpregnant individuals in which a higher intake of vegetables is associated with greater vaginal microbial diversity (p = 0.004) and *Lactobacillus crispatus* levels [9,16]. 

Dairy and Probiotics

Table 4 shows the pertinent findings of articles that analyze the relationship between dairy and probiotics and BV [17-22].

**Table 4:** Pertinent findings of studies describing the role of dairy and probiotics in BV. BV: bacterial vaginosis

Study	Pertinent findings
Fettweis et al. [17]	A negative correlation with BV-associated bacteria with greater yogurt consumption
Laue et al. [18]	The administration of a yogurt containing *Lactobacillus crispatus*, *Lactobacillus gasseri*, *Lactobacillus rhamnosus*, and *Lactobacillus jensenii* in addition to antibiotic treatment improved recovery rate and symptoms of BV in 100% of clinical trial group compared to only 76.5% in the placebo group
Happel et al. [19]	Unclear relationship between probiotic use and vaginal health, however stressed the role of influencing factors (e.g., smoking, lower income, etc.) on BV
Oerlemans et al. [20]	The samples from the probiotic group showed higher relative abundances of lactobacilli than non-probiotic groups (p = 0.04)
Gabriela et al. [21]	Women with vaginal microbiota dominated by *Lactobacillus iners* who consume milk and/or dairy present increased abundances of *Lactobacillus crispatus* which has protective properties of temporal microbiota stability and, consequently, increased protection against infections
Houttu et al. [22]	The vaginal microbiota composition was strengthened by the findings of a reduced abundance of potential pathobionts, namely, *Prevotella*, *Peptoniphilus*, *Dialister*, and *Campylobacter* from early to late pregnancy

Several studies have explored the role of probiotics and dairy products in reducing BV risk and improving outcomes by supporting a Lactobacillus-dominated vaginal microbiota [17-20]. An increased relative abundance of *L. crispatus* and *L. iners* has been reported to be higher for those with milk/dairy intake [21]. Previous literature has shown that *Lactobacillus* species with more *L. iners*-dominated samples, prevalent in probiotic groups, demonstrate a trend toward lower relative abundances of *Prevotella* [20]. Additionally, probiotic use reduced some potential pathobionts during pregnancy [22]. Overall, a trend toward reduced BV risk is demonstrated with greater yogurt consumption [17,18]. Though there may be an unclear relationship between probiotic use, these authors suggested other influencing factors, such as smoking and lower income, pointing to the multifactorial nature of BV [19].

Carbohydrates and Sugar (High Glycemic Index)

Table 5 shows the pertinent findings of articles that analyze the relationship between carbohydrates and sugar and BV [12,23-25].

**Table 5:** Pertinent findings of studies describing the role of carbohydrates and sugar in BV. DGI: dietary glycemic index; DGL: dietary glycemic load; BV: bacterial vaginosis

Study	Pertinent findings
Noormohammadi et al. [12]	Sugar-sweetened beverages and fruit juice, trans fatty acids, and sodium intake were directly associated with bacterial vaginosis odds
Noormohammadi et al. [23]	In this hospital-based case-control study, greater adherence to high-DGI/DGL diet and a low-fiber diet was significantly associated with increased odds of BV after adjustment for other covariates
Dall'Asta et al. [24]	Obesity and higher BMI were linked to a shift towards vaginal dysbiosis and higher Nugent scores, suggesting an imbalance in the vaginal microbiota (p = 0.034)
Miller et al. [25]	Greater carbohydrate intake was associated with a higher abundance of *Lactobacillus crispatus*, while lower carbohydrate intake trended toward more *Lactobacillus iners* and anaerobic species (p = 0.056)

Studies have aimed to speculate the role of carbohydrates and sugars in influencing BV risk, which appears to vary based on the population studied [12,23-25]. Studies performed on non-pregnant individuals show that a high dietary glycemic index (DGI) and dietary glycemic load (DGL), measures of carbohydrate and sugar intake, have been shown to significantly increase the risk of BV (p = 0.003 and 0.029, respectively) [12,23]. One study showed that sugar-sweetened beverages and fruit juice intake were directly associated with BV odds [12]. Conversely, specific studies on pregnant populations have shown that a higher intake of carbohydrates and sugars seemed to be associated with a lower Nugent score (a standardized Gram stain-based scoring system to diagnose BV) and a *Lactobacillus*-dominated vaginal flora, indicative of good vaginal health [24,25].

Grains and Fiber

Table 6 shows the pertinent findings of articles that analyze the relationship between grains and fiber and BV [9,23,24,26,27].

**Table 6:** Pertinent findings of studies describing the role of grains and fiber in BV. BV: bacterial vaginosis

Study	Pertinent findings
Rosen et al. [9]	Increased intake of fiber associated with increased *Lactobacillus crispatus* in the vaginal microbiome and statistical heterogeneity
Noormohammadi et al. [23]	A low dietary fiber diet was associated with an increased risk of BV after adjustment of potential confounders
Dall'Asta et al. [24]	A higher intake of fiber was found to be associated with a lower risk of BV
Sun et al. [26]	Pregnant women with a higher intake of grains demonstrated a trend of decreased alpha diversity of the vaginal microbiome with an increase of *Lactobacillus*-dominant bacteria
Shivakoti et al. [27]	An inverse relationship was found between a diet rich in fiber and BV odds

In addition to fruits and vegetables, numerous studies have shown that grains and fiber have a significant role in the risk and management of BV [9,23,24,26,27]. Specifically, a 75% increase in whole-grain intake in the diet corresponded to a higher *Lactobacillus*-dominant vaginal microbiota, thus lowering the risk of BV [26]. Moreover, there is an association between increased fiber intake and decreased risk of BV, with odds ratios of 0.22 and 0.49, and vice versa, as fiber promotes a balanced vaginal microbiome [9,23,24,27].

Protein

Table 7 shows the pertinent findings of articles that analyze the relationship between protein and BV [8,15,23,24].

**Table 7:** Pertinent findings of studies describing the role of protein in BV. BV: bacterial vaginosis

Study	Pertinent findings
Noormohammadi et al. [8]	Unhealthy diet pattern including the consumption of red meats had a 2.04 higher chance for BV
Noormohammadi et al. [15]	Participants in the last tertile of meat consumption in the alternative healthy eating index had lower odds of BV
Noormohammadi et al. [23]	Participants in the last tertile of meat consumption in the alternative healthy eating index had lower odds of BV
Dall'Asta et al. [24]	Higher intake of animal-sourced protein was associated with an increased risk of BV during pregnancy

Numerous studies have demonstrated that a *Lactobacillus*-dominated vaginal microbiota is negatively associated with higher pre-pregnancy consumption of animal-sourced protein. This association emphasizes the benefits of plant-based protein and fiber-rich diets in reducing BV risk [15,23,24]. Diets high in red meat are associated with increased BV risk, emphasizing plant-based patterns as a potential dietary modification in BV prevention [8].

Diet

Table 8 shows the pertinent findings of articles that analyze the relationship between diet and BV [8,11,23].

**Table 8:** Pertinent findings of studies describing the role of diet in BV. BV: bacterial vaginosis

Study	Pertinent Findings
Noormohammadi et al. [8]	Unhealthy diet pattern comprising of sugar, solid oils, sweets and desserts, red meat, fried potato, refined grains, visceral meat, and sweet drinks, had a 2.04 higher chance for BV
Song et al. [11]	Participants following a vegetarian diet exhibited higher average vaginal microbial diversity than nonvegetarians
Noormohammadi et al. [23]	Plant-based dietary patterns lower BV odds

Some studies have assessed the benefits of a plant-based diet in demonstrating a protective effect against BV due to decreased BV-associated bacteria and increased beneficial bacteria in the vaginal microbiome [11,23]. One study showed that women who ate an ovo-vegetarian diet, which consisted of all kinds of vegetables, beans, whole grains, and eggs, had an 84% lower chance of experiencing BV [8]. In a similar study, participants following a vegetarian diet exhibited higher average vaginal microbial diversity than nonvegetarians, lowering their risk for infection [23].

BMI

Table 9 shows the pertinent findings of articles that analyze the relationship between BMI and BV [8,11,14,15,24].

**Table 9:** Pertinent findings of studies describing the role of BMI in BV. BV: bacterial vaginosis; BMI: body mass index

Study	Pertinent findings
Noormohammadi et al. [8]	Compared to the healthy women, BV-affected women tended to have higher levels of obesity (p = 0.016)
Song et al. [11]	Obesity was associated with increased odds of BV
Akoh et al. [14]	In the teen cohort, higher pre-pregnancy BMI was associated with increased risk of BV
Noormohammadi et al. [15]	BV cases had a substantially higher median BMI than healthy controls
Dall'Asta et al. [24]	Higher BMI was associated with a lower poorer vaginal status

Numerous studies have indicated a positive correlation between elevated BMI and obesity with risk for BV in both pregnant and non-pregnant individuals [8,11,14,15,24]. One study, in particular, identified a statistically significant higher level of obesity in BV-affected women compared to healthy women (p = 0.016) [8].

Discussion

BV is one of the most common vaginal infections affecting several people globally. This condition is triggered by the imbalance of the vaginal microbiota. Disease typically occurs due to the overpopulation of *Gardnerella* species in an environment typically predominated by lactobacilli species. Given its lasting implications on reproductive and physical health, its association with diet is vital to study. Primary care physicians and physicians of obstetrics and gynecology (OB-GYN) should take the time to educate their young female patients on vital dietary modifications to protect their healthy vaginal microbiome from disease.

Based on the analysis of this systematic review, deficiencies of vitamins A, C, E, and D can increase the odds of procuring BV [7-10]. Vitamin D plays a significant role in promoting vaginal epithelial cell growth, which functions to improve the barrier nature of the vagina [28]. Vitamin D supplementation has been shown to benefit patients with atrophic vaginitis, suggesting that its incorporation in the diet might be beneficial for avoiding BV through improved barrier mechanisms [29]. Given the growing evidence linking vitamin D sufficiency to improved vaginal epithelial integrity and reduced infection risk, primary care providers and OB-GYNs should routinely assess vitamin D status in patients with recurrent BV as well as high-risk groups such as Black women and those with limited sun exposure. Furthermore, vitamins A, C, and E are known antioxidants, and they may play a role in reducing the oxidative stress in the vaginal microbiome, thus enhancing immunity against the pathogens that may cause BV [30]. Along a similar vein, beta-carotene, a pro-vitamin A, similarly renders immunity against pathogens via an antioxidative effect [31]. Additionally, this review identified the potential benefits of the consumption of betaine and other dietary phytochemicals [10,11,13]. While their specific role is unclear, the hypothesis is that betaine plays a role in the survival and growth of lactobacilli species by promoting the bacteria’s osmotolerance [10].

Moreover, our findings suggest an association between increased consumption of fruits and vegetables with a reduced risk of BV [7-13]. The benefit of fruits and vegetables in association with BV can be postulated due to the high content of antioxidants, vitamins, and phytochemicals, which contribute to and support the vaginal microbiota [13]. Based on this evaluation, specific fruits and vegetables high in vitamins A, C, E, D, and betaine can be recommended to lower the risk of BV. Sources of beta-carotene, or vitamin A, include carrots, sweet potatoes, butternut squash, and dark leafy greens [32]. To ensure adequate levels of vitamin C, at-risk individuals should consume citrus fruits, bell peppers, and strawberries [33]. Sources of vitamin E include avocado, spinach, swiss chard, and beet greens [34]. Although most fruits and vegetables do not contain adequate amounts of vitamin D, mushrooms contain high levels [35]. In addition to sources of vitamin-rich fruits and vegetables, beets and spinach are abundant sources of betaine [13]. Overall, our findings underscore the importance of a diet high in fruits and vegetables to lower the risk of BV due to the implications of a low vitamin and mineral diet in terms of vaginal health. Encouraging individuals to “eat the rainbow” by consuming a wide variety of colorful fruits and vegetables is a practical strategy to enhance intake of essential vitamins, antioxidants, and phytochemicals that support vaginal health. Nutrient synergy can further enhance these effects; for example, the absorption of fat-soluble compounds such as beta-carotene and vitamin E is improved when consumed alongside healthy fats such as avocado or olive oil. These nutrients collectively help reduce oxidative stress, modulate inflammation, and strengthen epithelial and immune function, factors critical to maintaining a balanced vaginal microbiome. Preparing vegetables with herbs and spices such as turmeric, oregano, and ginger may further support anti-inflammatory pathways. Emphasizing diverse, plant-forward meals that include both raw and cooked forms of fruits and vegetables can help optimize nutrient variety and bioavailability. In line with these strategies, the World Health Organization and Food and Agriculture Organization recommend a daily consumption of at least 400 g of edible fruits and vegetables, equivalent to about five servings of 80 g each as a population-wide target to prevent noncommunicable diseases and micronutrient deficiencies [36]. Overall, our findings underscore the importance of a diet high in fruits and vegetables to lower the risk of BV due to the implications of a low vitamin and mineral diet in terms of vaginal health.

Carbohydrate and sugar intake appear to have a strong correlation with increasing BV risk according to the studies in this review [12,23-5]. This is because foods with higher glycemic index and load tend to cause increased oxidative stress in the body, thereby suppressing immune support from the prevention of disease [12]. However, the results of this review show a converse relationship between carbohydrates and pregnant populations. Pregnant women with a higher intake of carbohydrates and sugars demonstrated healthier vaginal microbiota and vaginal health [24,25]. Lactobacilli species in the vagina ferment carbohydrates into simple sugars, producing lactic acid during the fermentation process. This results in a lower pH in the vaginal environment, which restricts the growth of bacteria such as *Gardnerella vaginalis* that tend to be sources of BV infection [35]. This review hypothesizes that the difference in the relationship between carbohydrates and BV in pregnant and non-pregnant populations can be explained by the increased energy demand for both maternal and fetal health in pregnancy. Changes to maternal hormone secretions as well as fetal nutrient supply result in increased metabolism of carbohydrates in pregnant women compared to non-pregnant women, resulting in increased lactic acid concentrations and thus a more protective vaginal environment [37]. The results of this review thus lead us to recommend that pregnant women increase their carbohydrate intake, and that non-pregnant women consume smaller quantities of carbohydrates to protect from BV infection.

A higher intake of whole grains and dietary fiber decreases the risk of BV due to the promotion of a *Lactobacillus*-dominant vaginal microbiota [9,23,24,26,27]. Dietary fiber serves as a prebiotic substrate, promoting beneficial gut bacteria that produce short-chain fatty acids (SCFAs) such as butyrate. These SCFAs help regulate inflammation and strengthen mucosal barriers, influencing not only gut but also vaginal microbial health [38]. Whole grains contain fiber and polyphenols that have been shown to lower inflammatory markers in the body [39]. With this, diets high in whole grains and dietary fiber can reduce systemic inflammation associated with BV to maintain a balanced vaginal microbiota by modulating gut microbiota, lowering oxidative stress, and improving insulin sensitivity [23]. SCFAs generated from fiber fermentation contribute to lowering systemic inflammation and improving insulin sensitivity, which supports a vaginal environment favorable to *Lactobacillus* species dominance and reduced BV risk [38]. Moreover, whole grains also contain essential nutrients such as zinc, magnesium, and B vitamins, which play a role in the immune system [40]. Specifically, zinc increases IL-2 production and supports Th1 responses, magnesium supports the regulation of pro-inflammatory cytokines like interleukin (IL)-6 and tumor necrosis factor-alpha, and B vitamins, particularly B6, influence IL-2 production and lymphocyte proliferation, while folate and B12 support DNA synthesis in immune cells. Together, these nutrients coordinate to enhance immune responses and maintain mucosal defenses essential for a balanced vaginal microbiome. Due to this, the indirect role of whole grains in immune system support suggests the maintenance of the vaginal microbiota and resistance imbalances [41].

Based on the results of this review, a diet rich in fiber and whole grains is recommended to prevent BV. Whole-grain foods high in fiber and B vitamins include quinoa, brown rice, oats, millets, and whole-wheat bread/pasta, as well as other whole grains commonly consumed in diverse cultural diets [42]. Consuming a variety of fiber types, including soluble and insoluble fibers from legumes, whole grains, and seeds, supports diverse gut bacteria and sustains SCFA production vital for immune regulation. In addition, legumes such as lentils and chickpeas are rich in prebiotic fiber [43]. For further anti-inflammatory effects, at-risk individuals should consume flaxseeds and chia seeds, which are sources of soluble fiber [44].

This study identified that all women with elevated BMI, which is typically associated with overweight states and obesity, have an increased odds of acquiring BV infection [8,11,14,15,24]. Obesity is considered a chronic inflammatory state itself, and results in increased secretion of pro-inflammatory proteins, such as leptin, from the greater concentration of adipose tissue in an obese individual. While this mechanism is not fully understood, it is hypothesized that these alterations in the immune system likely create an unfavorable environment for lactobacilli species in the vagina [45].

Building on the discussion of strategies for improving women’s health, it is vital to understand the impact of dietary choices, particularly dairy and probiotics, on the prevention and management of BV and its associated outcomes. This review highlights multiple studies that have examined how probiotics and dairy products influence BV risk by fostering a vaginal microbiota dominated by *Lactobacillus* species [17-20]. During the fermentation of dairy products, the process of proteolysis breaks down milk proteins, thereby releasing bioactive peptides. These peptides, along with the probiotics present, contribute to gut microbiota balance and support overall health [46]. Specifically, the vaginal ecosystem, lactobacilli strains help maintain this balance by producing lactic acid, which lowers vaginal pH and inhibits organisms that may cause BV [30]. While these lactobacilli are unsuitable as primary fermentation starters for industrial dairy production, certain strains of *L. crispatus* and *L. gasseri* maintain high viability in pasteurized milk. This suggests their potential as adjunct cultures in developing functional dairy products tailored toward women’s health, representing a step toward innovation in the dairy industry [47]. This also underscores the potential for probiotics to serve as a targeted strategy to modulate the vaginal microbiota, reducing harmful bacteria and possibly lowering the risk of BV. Additionally, incorporating these probiotic strains into dietary recommendations could enhance preventive and therapeutic approaches for maintaining vaginal health.

There is also evidence that probiotics help reduce the presence of certain harmful bacteria during pregnancy, further supporting their potential health benefits [22]. Specifically, increased yogurt intake has been associated with a lower likelihood of developing BV [17,18]. Such dietary recommendations may serve as practical adjuncts to other prevention strategies. However, despite these associations, some researchers emphasize that BV risk is influenced by multiple factors. For instance, smoking and lower socioeconomic status may also contribute to BV risk [19]. This points to the need for a comprehensive approach to BV prevention that considers not only dietary and probiotic interventions but also socioeconomic and lifestyle factors, such as income, education, employment opportunities, and access to quality healthcare.

Shifting the focus to another dietary component, it is also vital to examine the role of protein intake and its potential impact on the prevention and outcomes of BV. Plant-based proteins may offer distinct advantages for vaginal microbiota compared to animal-based proteins due to their effects on pH balance, inflammation, and bacterial composition. High-protein diets, particularly those rich in animal proteins, contribute to an increased dietary acid load, which can disrupt systemic pH balance and may influence vaginal acidity, which is an essential factor in the development of BV [12]. Conversely, plant-based foods, which are rich in fiber and alkaline precursors, help maintain a more favorable vaginal pH, therefore making the vaginal environment less likely for the development of BV. Additionally, plant-based diets are abundant in flavonoids, known anti-inflammatory agents which inhibit NF-kB activation, thereby inhibiting pro-inflammatory pathways linked to BV [48,49]. Given that inflammation and pH dysregulation are central to BV pathogenesis, shifting towards plant-based protein sources may foster a healthier vaginal microbiome by reducing acidity and inflammation, thereby lowering BV risk.

The above-mentioned recommendations to consume a diet rich in fruits, vegetables, legumes, whole grains, fiber, dairy, and plant-based protein to protect from BV align with the data in this review that show the protective nature of a plant-based or vegetarian diet [8,11,12]. Given the multinational prevalence of BV and the accessible nature of the components of this recommended diet, these recommendations stand for all populations globally. Furthermore, higher scores on global diet quality indices are strongly associated with reduced BV risk, emphasizing that overall dietary patterns, rather than individual nutrients alone, are critical for maintaining vaginal and reproductive health [50]. Encouraging diverse, nutrient-dense diets supports a balanced vaginal microbiome and broader systemic well-being.

Limitations

While this review hypothesizes the way dietary nutrients affect vaginal health, further studies must be performed to truly understand these specific mechanisms and determine dose-dependent relationships to determine optimal intake of each dietary nutrient. Several limitations should be considered when interpreting the results. These constraints highlight the need for further research to address potential gaps. Furthermore, it is important to note that this review utilized articles from three databases only, suggesting that there may be more existing research available on databases not explored by this review.

One study determined a borderline significant association of some minerals, such as zinc, selenium, and lutein, with BV, and further studies are essential to ascertain their specific relationship [10]. Furthermore, this review was unable to postulate the efficacy of these dietary nutrients against each other, as this type of data is currently unavailable. Additionally, there is a need for longitudinal studies that assess dietary patterns over time to determine causality rather than mere correlation, and would also enable the control of confounding variables that may affect both diet and microbiota composition. These considerations underscore the importance of an integrative approach to dietary recommendations, where nutritional guidance is tailored to promote reproductive tract health.

Further studies are also required to determine how these dietary recommendations might have to be altered for populations based on age range, gender, ethnicity, and even sociocultural practices. For example, a study found an association between an increased intake of fruits and increased *L. crispatus* in the vaginal microbiome only in Black women [9]. More studies specifically altered for diverse populations would be beneficial in making specific dietary recommendations. With the same idea, BV is associated with many comorbidities, and this review fails to identify and explain the diet modifications for individuals with BV who are also suffering from these comorbidities. Moreover, the studies in this review demonstrated promising results; however, many limitations, such as small sample sizes and a shortage of longitudinal studies, need to be addressed in further studies to address the lack of regulatory approval and clinical validation.

## Conclusions

The findings of this review highlight the influential role diet plays in BV susceptibility for both pregnant and non-pregnant women around the world. Given BV’s high prevalence and lack of management strategies, educating patients on their nutritional intake is vital for overall health. Therefore, physicians and healthcare providers must incorporate conversations about nutritional status with at-risk patients. Increasing intake of nutrients such as plant-based proteins, whole grains, and dietary fibers may help women lower their risk of BV.

## References

[1] Onderdonk AB, Delaney ML, Fichorova RN (2016). The human microbiome during bacterial vaginosis. Clin Microbiol Rev.

[2] Kairys N, Carlson K, Garg M (2024). Bacterial Vaginosis. StatPearls.

[3] Bautista CT, Wurapa E, Sateren WB, Morris S, Hollingsworth B, Sanchez JL (2016). Bacterial vaginosis: a synthesis of the literature on etiology, prevalence, risk factors, and relationship with chlamydia and gonorrhea infections. Mil Med Res.

[4] Coudray MS, Madhivanan P (2020). Bacterial vaginosis-a brief synopsis of the literature. Eur J Obstet Gynecol Reprod Biol.

[5] Mondal AS, Sharma R, Trivedi N (2023). Bacterial vaginosis: a state of microbial dysbiosis. Med Microecol.

[6] Tricco AC, Lillie E, Zarin W (2018). PRISMA Extension for Scoping Reviews (PRISMA-ScR): checklist and explanation. Ann Intern Med.

[7] Bisanz JE, Enos MK, PrayGod G (2015). Microbiota at multiple body sites during pregnancy in a rural Tanzanian population and effects of Moringa-supplemented probiotic yogurt. Appl Environ Microbiol.

[8] Noormohammadi M, Eslamian G, Kazemi SN, Rashidkhani B (2022). Association between dietary patterns and bacterial vaginosis: a case-control study. Sci Rep.

[9] Rosen EM, Martin CL, Siega-Riz AM (2022). Is prenatal diet associated with the composition of the vaginal microbiome?. Paediatr Perinat Epidemiol.

[10] Tuddenham S, Ghanem KG, Caulfield LE (2019). Associations between dietary micronutrient intake and molecular-bacterial vaginosis. Reprod Health.

[11] Song SD, Acharya KD, Zhu JE, Deveney CM, Walther-Antonio MR, Tetel MJ, Chia N (2020). Daily vaginal microbiota fluctuations associated with natural hormonal cycle, contraceptives, diet, and exercise. mSphere.

[12] Noormohammadi M, Eslamian G, Kazemi SN, Rashidkhani B (2022). Dietary acid load, alternative healthy eating index score, and bacterial vaginosis: is there any association? A case-control study. BMC Infect Dis.

[13] Khademian A, Noormohammadi M, Moori MH, Makhtoomi M, Esmaeilzadeh S, Nouri M, Eslamian G (2024). The association between dietary phytochemical index and bacterial vaginosis risk: secondary analysis of case-control study. J Health Popul Nutr.

[14] Akoh CC, Pressman EK, Cooper E, Queenan RA, Pillittere J, O'Brien KO (2017). Prevalence and risk factors for infections in a pregnant adolescent population. J Pediatr Adolesc Gynecol.

[15] Noormohammadi M, Eslamian G, Kazemi SN, Rashidkhani B (2022). Is there any association between adherence to the Mediterranean diet and dietary total antioxidant capacity with bacterial vaginosis? Results from a case-control study. BMC Womens Health.

[16] Vargas-Robles D, Yap YR, Singha B (2024). Association of diet and inflammation with the vaginal microbiota of pregnant individuals with or without IBD. bioRxiv.

[17] Fettweis JM, Brooks JP, Serrano MG (2014). Differences in vaginal microbiome in African American women versus women of European ancestry. Microbiology (Reading).

[18] Laue C, Papazova E, Liesegang A (2018). Effect of a yoghurt drink containing Lactobacillus strains on bacterial vaginosis in women - a double-blind, randomised, controlled clinical pilot trial. Benef Microbes.

[19] Happel AU, Jaumdally SZ, Pidwell T (2017). Probiotics for vaginal health in South Africa: what is on retailers' shelves?. BMC Womens Health.

[20] Oerlemans E, Ahannach S, Wittouck S (2022). Impacts of menstruation, community type, and an oral yeast probiotic on the vaginal microbiome. mSphere.

[21] B Moura G, G Silva M, Marconi C (2023). Milk and dairy consumption and its relationship with abundance of Lactobacillus crispatus in the vaginal microbiota: milk intake and vaginal Lactobacillus. J Low Genit Tract Dis.

[22] Houttu N, Mokkala K, Saleem WT (2022). Potential pathobionts in vaginal microbiota are affected by fish oil and/or probiotics intervention in overweight and obese pregnant women. Biomed Pharmacother.

[23] Noormohammadi M, Eslamian G, Kazemi SN, Rashidkhani B, Malek S (2022). Association of dietary glycemic index, glycemic load, insulin index, and insulin load with bacterial vaginosis in Iranian women: a case-control study. Infect Dis Obstet Gynecol.

[24] Dall'Asta M, Laghi L, Morselli S (2021). Pre-pregnancy diet and vaginal environment in Caucasian pregnant women: an exploratory study. Front Mol Biosci.

[25] Miller C, Morikawa K, Benny P (2024). Effects of dietary quality on vaginal microbiome composition throughout pregnancy in a multi-ethnic cohort. Nutrients.

[26] Sun H, Yamada P, Paetow A (2022). A randomized controlled trial of the effects of whole grains versus refined grains diets on the microbiome in pregnancy. Sci Rep.

[27] Shivakoti R, Tuddenham S, Caulfield LE (2020). Dietary macronutrient intake and molecular-bacterial vaginosis: role of fiber. Clin Nutr.

[28] Jefferson KK, Parikh HI, Garcia EM (2019). Relationship between vitamin D status and the vaginal microbiome during pregnancy. J Perinatol.

[29] Li D, Zhang T, Yang H, Yang W, Zhang C, Gao G (2023). Effect of vitamin D on the proliferation and barrier of atrophic vaginal epithelial cells. Molecules.

[30] Chee WJ, Chew SY, Than LT (2020). Vaginal microbiota and the potential of Lactobacillus derivatives in maintaining vaginal health. Microb Cell Fact.

[31] Weber D, Grune T (2012). The contribution of β-carotene to vitamin A supply of humans. Mol Nutr Food Res.

[32] Meléndez-Martínez AJ, Mandić AI, Bantis F (2022). A comprehensive review on carotenoids in foods and feeds: status quo, applications, patents, and research needs. Crit Rev Food Sci Nutr.

[33] Chambial S, Dwivedi S, Shukla KK, John PJ, Sharma P (2013). Vitamin C in disease prevention and cure: an overview. Indian J Clin Biochem.

[34] Sinbad OO, Folorunsho AA, Olabisi OL (2019). Vitamins as antioxidants. J Food Sci Nutr Res.

[35] Cardwell G, Bornman JF, James AP, Black LJ (2018). A review of mushrooms as a potential source of dietary vitamin D. Nutrients.

[36] Rippin HL, Maximova K, Loyola E (2023). Suboptimal intake of fruits and vegetables in nine selected countries of the World Health Organization European region. Prev Chronic Dis.

[37] Williamson CS (2006). Nutrition in pregnancy. Nutr Bull.

[38] Takada K, Melnikov VG, Kobayashi R, Komine-Aizawa S, Tsuji NM, Hayakawa S (2023). Female reproductive tract-organ axes. Front Immunol.

[39] Khan J, Gul P, Rashid MT, Li Q, Liu K (2024). Composition of whole grain dietary fiber and phenolics and their impact on markers of inflammation. Nutrients.

[40] Slavin JL, Jacobs D, Marquart L, Wiemer K (2001). The role of whole grains in disease prevention. J Am Diet Assoc.

[41] Gombart AF, Pierre A, Maggini S (2020). A review of micronutrients and the immune system-working in harmony to reduce the risk of infection. Nutrients.

[42] Slavin J (2004). Whole grains and human health. Nutr Res Rev.

[43] Kadyan S, Sharma A, Arjmandi BH, Singh P, Nagpal R (2022). Prebiotic potential of dietary beans and pulses and their resistant starch for aging-associated gut and metabolic health. Nutrients.

[44] Kajla P, Sharma A, Sood DR (2015). Flaxseed-a potential functional food source. J Food Sci Technol.

[45] Garg A, Ellis LB, Love RL, Grewal K, Bowden S, Bennett PR, Kyrgiou M (2023). Vaginal microbiome in obesity and its impact on reproduction. Best Pract Res Clin Obstet Gynaecol.

[46] Widyastuti Y, Febrisiantosa A, Tidona F (2021). Health-promoting properties of lactobacilli in fermented dairy products. Front Microbiol.

[47] Siroli L, Patrignani F, Serrazanetti DI, Parolin C, Ñahui Palomino RA, Vitali B, Lanciotti R (2017). Determination of antibacterial and technological properties of vaginal lactobacilli for their potential application in dairy products. Front Microbiol.

[48] Serafini M, Peluso I, Raguzzini A (2010). Flavonoids as anti-inflammatory agents. Proc Nutr Soc.

[49] Kalia N, Singh J, Kaur M (2019). Immunopathology of recurrent vulvovaginal infections: new aspects and research directions. Front Immunol.

[50] Mehrabani S, Moori MH, Normohammadi M (2025). The association between global and prime diet quality scores and the risk of bacterial vaginosis: a secondary analysis of case-control study. J Health Popul Nutr.

